# The Consequence of the Presence of Ribonucleotide for ds-DNA’s Electronic Properties: Preliminary Theoretical Studies

**DOI:** 10.3390/cells14120881

**Published:** 2025-06-11

**Authors:** Boleslaw T. Karwowski

**Affiliations:** DNA Damage Laboratory of Food Science Department, Faculty of Pharmacy, Medical University of Lodz, ul. Muszynskiego 1, 90-151 Lodz, Poland; boleslaw.karwowski@umed.lodz.pl

**Keywords:** ribonucleotides, DNA, ionisation potential, electron affinity, DFT

## Abstract

The genome is continuously exposed to different harmful factors whose activity causes different types of lesions. On the other hand, during the DNA replication process, a ribonucleoside (rN) can be inserted more frequently than the occurrence of DNA damage in the genome. Notably, it can be expected that their presence and chemical lability change the electronic properties of the double helix. In this study, a short ds-oligo with a single rN was taken into consideration. The ground-state molecular geometry was obtained at the M06-2x/D95* level of theory in the aqueous phase, while the energy and vertical and adiabatic electron affinity and ionisation potential were obtained at M06-2x/6-31++G**. The obtained results indicate that the 3′,5′-phosphodiester bond cleavage is favourable after the adiabatic cation and anion states are achieved by ds-DNA. Moreover, the lowest ionisation potential and highest electron affinity of 2.76 and 5.55 eV, respectively, which make it a suitable endpoint for electrons and holes, have been found for the final product that contains a single-strand break. Additionally, after the internucleotide 3′,5′→2′,5′ bond migration process, proton-coupled electron transfer was found to occur. In this article, the electronic properties of short ds-DNA fragments with ribonucleoside inserts are reported for the first time. The obtained results suggest that rNs can play a significant role in the communication of repair and replication proteins via electron transfer, especially after rearrangement. Moreover, the discussed internucleotide bond stability changes after one-electron oxidation or reduction and can support new radiotherapy strategies that are safer and more effective. Further theoretical and experimental studies are highly warranted.

## 1. Introduction

**Figure 1:**
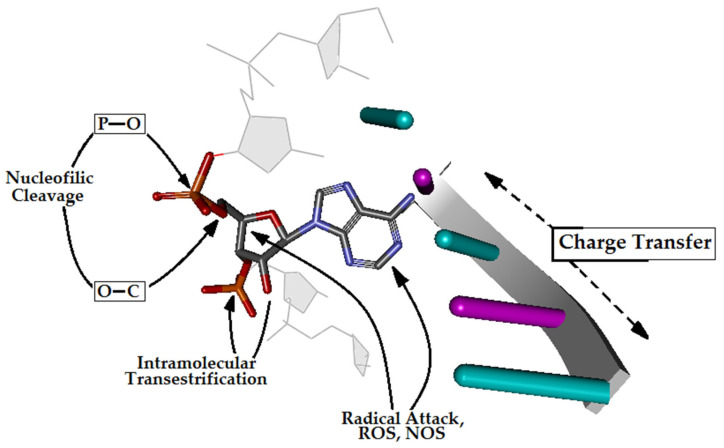
A graphical representation of RNA phosphate diester bond cleavage and possible pathways to nucleic acid injury, such as intramolecular transesterification, oxidative cleavage, and nucleobase modification in the cell environment.

The genome—the seed of life—is continuously exposed to different harmful extra- and intracellular factors, such as alkylating agents, reactive oxygen/nitrogen species (RO/NS), ionisation radiation, aromatic amines and polycyclic aromatic hydrocarbons [1]. Additionally, genomic DNA is subject to replication errors and topoisomerase misalignment [2]. As shown in Figure 1, different parts of DNA are susceptible to radical or nucleophilic cleavage or modification [3]. To date, more than 70 different types of damage have been described [4]. Notably, in each of the 10^14^ cells in the human body, approximately 2500 DNA-damaging events occur every hour [5]; most are related to single-strand breaks and depurination. Therefore, in the human body, approximately 3×10^17^ DNA lesions can appear every hour. Different repair systems maintain the stability of genetic information, such as base or nucleotide excision repair (BER, NER), homologous recombination (HR), non-homologous end-joining (NHEJ) or mismatch repair [6]. However, BER glycosylases are the front-line system proteins that recognise and excise damaged bases [7]. These proteins scan the double helix and utilise charge transfer for mutual communication to increase their efficiency [8]. It should be pointed out that the [Fe-S] cluster has been found not only in glycosylases, such as MutYh and Exonuclease III, but also in replicative enzymes (pol ε/δ) [9]. The above information indicates that proteins involved in DNA repair and replication use the double helix as a communicative nanowire [10].

**Figure 2:**
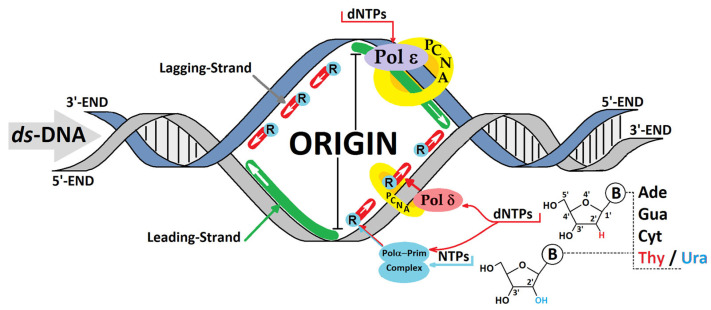
A schematic action overview of the replicative polymerases (Pols) during leading- and lagging-strand replication. The possibilities for ribonucleotide incorporation by the polymerases Pol α, δ, and ε with different frequencies. PCNA—proliferating cell nuclear antigen, dNTP—2′-deoxyribonucleotide triphosphate, NTP—ribonucleoside triphosphate, R—ribonucleotide.

Electron or hole transfer through the double helix is sensitive to the spatial arrangement of nucleobases, such as heterocyclic ring overlapping (stacking) and hydrogen bonding [11]. Their disruption can lead to serious consequences during hole hopping, polaron migration and tunnelling. Charge transfer can be affected not only by DNA lesions, such as 8-oxo-Gua, and pyrimidine dimers, such as T^T, but also by mismatches. High-fidelity polymerases (replicases) can incorporate an incorrect nucleoside at a frequency of one per thousand during genetic information replication [12,13]. Moreover, if the concentration of ribonucleoside triphosphates (NTPs) is higher than that of deoxyribose (dNTPs) in the cell environment, the misincorporation of nucleotides with different sugar moieties is much more common [12]. It has been estimated that, even with a sterically clashing polymerase active centre, pol ε/δ (replicase) incorporates 1 NTP per 1250–5000 nucleotides [14]. Meanwhile, for pol α, this figure has been found to be 1 per 625 bases due to the lack of an exonuclease subunit [15,16]. This indicates that more ribonucleosides appear during lagging-strand synthesis through Okazaki fragments, where pol α–primase complex activity is present (Figure 2) [17]. In one replication cycle, 13,000 ribonucleotides can appear in the yeast genome [15] and approximately 10^6^ in vertebrate cells [18]. The ribonucleotide part is removed (repaired) from the genome by the ribonucleotide excision repair (RER) machinery [19,20].

Although the effects of modified bases, single-strand breaks and cross-links on the charge transfer through the double helix have been investigated experimentally and theoretically, the role of ribonucleosides has not yet been examined. Here, for the first time, the influence of a ribonucleotide subunit present in ds-DNA on its electronic properties is theoretically considered and discussed.

## 2. Materials and Methods

**Figure 3:**
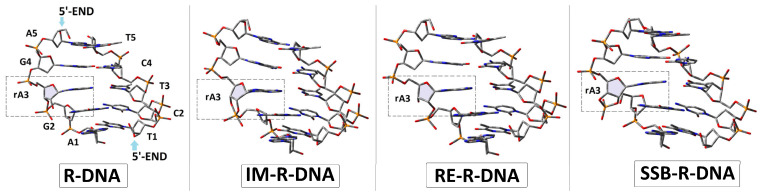
The optimised at the M06-2x/D95* level of theory in the aqueous phase spatial geometry of the ds-oligonucleotides discussed in the article. The dotted square indicates the location of Adenine (ribonucleoside) in the double-stranded DNA structure.

The initial structures of the double-stranded oligonucleotide with a ribonucleoside moiety (adenosine) were built using the BioVia Discovery Studio v20.1.0.19295 software [21] and noted as follows: R-DNA d[T_1_C_2_T_3_C_4_T_5_]*d[A_5_G_4_]rA_3_[G_2_A_1_], RE-R-DNA d[T_1_C_2_T_3_C_4_T_5_]*d[A_5_G_4_]X_3_[G_2_A_1_] (X: 2′,5′-phosphodiester linkage), IM-R-DNA d[T_1_C_2_T_3_C_4_T_5_]*d[A_5_G_4_]Z_3_[G_2_A_1_] (Z: the pentacoordinated species, called pentaoxyphosphorane), and SSB-R-DNA d[T_1_C_2_T_3_C_4_T_5_]*d[A_5_G_4_]Y_3_[G_2_A_1_] (Y: 2′,3′-cyclic-phosphate). The optimised spatial structures of the ds-oligos are shown in Figure 3.

The negative charges of the phosphate groups were neutralised by the addition of protons, and the other atoms were saturated by additional hydrogen atoms as necessary. The spatial structure *ds*-pentamers were optimised according to the QM/MM methodology. The short double-stranded pentamers were divided into two levels of calculation using the ONIOM (Our own N-layered Integrated Molecular Orbital and Molecular Mechanics) method, i.e., high level, M06-2X/D95*-HL (all the nucleobases and the whole nucleotide dimer dA3_PO2_G4), and low level, M06-2X/sto-3G-LL (the remain sugar–phosphate backbones) [22,23,24,25].

All the energies discussed in this article were obtained in the condensed (aqueous) phase using Tomasi’s Conductor-like Polarised Continuum Model (C-PCM). For this propose, the Minnesota functional, M06-2x, with an augmented polarised valence double-ζ basis set 6-31++G** for complete oligonucleotides structure was applied [26,27,28]. For the extracted fragment from the parent ds-oligo structure, the spatial locations of the hydrogen atoms added for saturation were optimised (M06-2X/D95* level of theory in the condensed phase), with the position of all the other atoms frozen [29,30]. For all the discussed molecules, energy, charge and spin distribution analysis was achieved using the Hirshfeld methodology at the M06-2X/6-31++G** level of theory in the condensed phase [31]. The ds-oligonucleosides’ electronic properties were calculated as described previously; please see reference [29,30,31,32]. The solvent–solute interaction was looked at in the non-equilibrium (NE) and equilibrated (EQ) modes [33]. The energy of the molecules in the NE mode was calculated using two-step processes according to the save–read procedures implemented in the Gaussian G16 software package. The following energy notation was applied, i.e., the *E*_geometry_^charge^ of the molecule (neutral form) is described as *E***_0_^0^**, the vertical cation/anion as *E***_0_^+^**/*E***_0_**, the adiabatic cation/anion as *E***_+_^+^**/^+^*E***_−_^−^**, and the vertical neutral formed from the cation/anion state as *E***_0_^+^**/*E***_0_^−^**. The energy difference, in [eV], between the mentioned energies corresponds to the suitable electronic states described as follows: ^NE^VIP = *E*_0_^+(NE)^ − *E*_0_^0^ (Vertical Ionisation Potential in the NE state); ^EQ^VIP = *E*_0_^+(EQ)^ − *E*_0_^0^ (Vertical Ionisation Potential in the EQ state); AIP = *E_+_*^+^ − *E*_0_^0^ (adiabatic ionisation potential); ^NE^VEA = *E*_0_^−(NE)^ − *E*_0_^0^ (Vertical Electron Affinity in the NE state); ^EQ^VEA = *E*_0_^−(EQ)^ − *E*_0_^0^ (Vertical Electron Affinity in the EQ state); AEA = *E*_0_^0^ − *E*_−_^−^ (adiabatic electron affinity). All the above calculations were performed with the Gaussian G16 (version C.01) software package [34].

## 3. Results and Discussion

### 3.1. The Mechanistic Aspect or Internucleotide Phosphodiester Bond Cleavage and Migration

**Figure 4:**
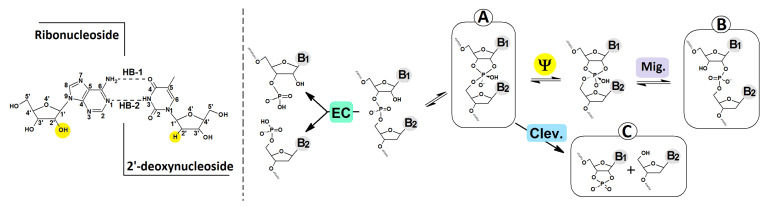
The possible path of RNA internucleotide phosphodiester bond rearrangement. Enzymatic cleavage (EC), SN_2_P cleavage (Clev.), 3′,5′→2′,5 phosphodiester linkage migration (Mig.) via Berry pseudorotation (Ψ). In the left scheme, the overview of the nucleoside pair with the atom numbering and hydrogen bond indication, i.e., HB-1: Adenine N6/Thymine O4 and HB-2: Adenine N1/Thymine N3.

The insertion of ribonucleotides into the ds-DNA structure can have several geometrical consequences, including changes in elasticity, deoxyribose puckering, minor groove width, base pair hydrogen bonding and duplex unwinding. Hence, to emphasise their effects on the charge distribution and electronic properties, the central deoxyadenosine of the initial native short double-stranded 2′-deoxyoligonucleotide d[AGAGA]*d[TCTCT] was substituted by riboadenosine, leaving the complementary thymidine unchanged. The above approach corresponds well with the situation where a nucleotide with the wrong sugar is introduced during the replication process. The concentrations of ATP and dATP in human peripheral blood monocytes have been found to be 6719 μM and 1.5 μM, respectively [35], which can support the frequent incorporation of ribonucleosides into ds-DNA. Moreover, RNA—and, therefore, oligonucleotides with ribonucleotide insertion—is less stable than DNA. The estimated half-life of the phosphodiester bonds in DNA is 30 million years, whereas, in RNA under favourable conditions (pH~7 and T~25 °C), it is just 10 years. Enzymes can accelerate this process by factors of 10^17^ and 10^11^, respectively. As shown in Figure 4, the RNA phosphodiester bond can be cleaved by proteins or via intramolecular 2′-OH attack. For the mechanistic details, please see the valuable review article by Lönnberg [36].

The nucleophilic attack of the ribose 2′-OH hydroxyl group at the internucleotide bond causes trigonal bipyramid formation with a pentacoordinated P atom at the central point. The two ligands present at the corners of the newly created structure occupy the apical position, while the other is equatorial. According to Westheimer’s rules, a nucleophile can enter and depart from the apical position [37]. If the formed intermediate of the SN_2_P reaction is stable enough, the Berry pseudorotation (Ψ) process can take place, and it has been almost exclusively used for RNA internucleotide phosphodiester bond cleavage or migration (Figure 4) [38,39]. Notably, in the intermediate structure, electronegative ligands occupy the apical position; moreover, the 3′ and 2′ oxygens bridged by the ethylene group adopt the equatorial and apical positions.

In this study, the structure of the double helix was taken into consideration instead of the simple single-strand fragment or base pair system. It should be noted that the spatial geometry of the double helix is stabilised by stacking interactions and hydrogen bonds. The proposed approach allowed for an investigation of the susceptibility of the internucleotide diester bond between ribo- and 2′-deoxyucleosides to cleavage or migration during positive or negative charge migration through ds-DNA. Therefore, the influence of a hole or extra electron present in the system on this process was considered.

**Figure 5:**
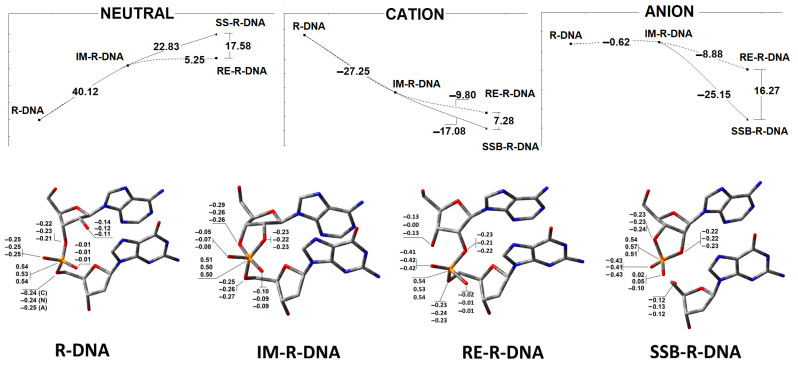
Upper graphs represent the energy profile of the phosphodiester bond cleavage or migration via an intermediate state in the neutral, anion and cation forms of the discussed ds-oligonucleotides. Botton: The charge distribution on atoms involved in the phosphodiester linkage. The results have been obtained at the M062x/6-31++G** level of theory in the aqueous phase using the Hirshfeld methodology. The raw data are given in Supplementary Materials Table S1.

As shown in Figure 4, in the neutral form of R-DNA, the nucleophilic attack of O2′ on phosphorus with the subsequent intermediate formation of IM-R-DNA is energetically unfavourable, with a calculated 40.12 kcal barrier. Moreover, further trigonal bipyramidal structure rearrangements toward P-O5′ bond cleavage or P-O3′→P-O2′ migration require an additional 22.83 and 5.25 kcal, respectively. The above results coincide well with experimental data, which have shown that, under neutral conditions (i.e., pH~7), the internucleotide bond formed by ribonucleotides is moderately stable in comparison to DNA. This observation is in good agreement with a previous theoretical analysis, which indicated that the barrier to endocyclic P-O3′ or P-O2′ bond cleavage was more than 10 kcal lower than that to exocyclic P-O5′ cleavage [40]. The extra electron appearing in the R-DNA causes radical anion formation (i.e., one-electron reduction). It has been found that the energetic difference between R-DNA and IM-R-DNA (in their anionic forms) slightly favours intermediate formation (−0.62 kcal) in the above-mentioned SN_2_P reaction. However, further bipyramidal pseudorotation leads to the dissociation of the bridged P-O5′ bond (SSB-R-DNA) being favoured (−25.15 kcal) over internucleotide bond migration (RE-R-DNA) (−8.88 kcal). Conversely, the electron loss by R-DNA causes radical cation formation (one-electron oxidation). At this stage, intermediate (IM-R-DNA) formation is favoured (−27.26 kcal). From an energetic point of view, further P-O3′→P-O5′ phosphodiester migration with the subsequent RE-R-DNA formation (via intermediate pseudorotation) has been calculated as less favourable (i.e., −9.8 kcal) than exocyclic P-O5′ linkage cleavage (i.e., −17.08 kcal) (Figure 5).

The above-discussed results are focused on the energy change of internucleotide bond migration or cleavage for a complete double-stranded pentamer with a single ribonucleoside inserted at its central point; namely, adenosine (A3). As the discussed process can take place exclusively at the ribonucleotide moiety, the charge distribution on atoms involved in P-O bond cleavage or migration was investigated at the M062x/6-31++G** level of theory in the aqueous phase using the Hershfield methodology. In Figure 5, the charge distribution is shown. In the neutral, anionic and cationic states, the O3′ atom has higher electronegativity than O2′ and adopts an apical position in the structure of the trigonal bipyramid. Moreover, in each case, the P-O5′ bond is found to be the longest one, 1.77 [Å], in the cyclic phosphorane intermediate IM-R-DNA (Table 1). P-O2′ and P-O5′ are found to be 0.02 and 0.03 [Å] shorter, respectively, when the neutral and anionic states of the ds-oligo are analysed and become significantly reduced when IM-R-DNA adopts the cation radical state after one-electron oxidation (i.e., 1.66 [Å], compared to 1.74 [Å] for P-O3′).

Phosphodiester bond migration via IM-R-DNA reorganisation (Figure 3) or via exocyclic P-O5′ cleavage leads to the RE-R-DNA and SSB-R-DNA products, respectively. The main structural difference from the parent R-DNA is P-O3′ or P-O5′ bond fission and P-O2′ formation, as shown in Table 1, independently of any electron loss or gain by the ds-oligo. These structural changes are compensated for by the ribose ring flexibility, which manifests in puckering and phase changes [41] (Table 1).

Moreover, the discussed phosphodiester bond rearrangement did not significantly affect the hydrogen bond length between adenosine (A3) and the complementary thymidine (T3) in the neutral state of all the discussed oligos, except for SSB-R-DNA, for which significant HB-2 shortening to 2.65 [Å] was noted. Similarly, this rearrangement was observed after electron loss by the ds-oligo; however, in the case of R-DNA, shorter HB-1 (2.85 [Å]) and HB-2 (2.66) [Å] were calculated. Surprisingly, introducing an extra electron into the system did not affect HB-1 at all, while a shorter HB-2 was observed for IM-R-DNA (2.88 [Å]). As shown in Table 1, the fluctuations of HB-1/HB-2 were assigned in the length ranges of 0.1/0.23, 0.17/0.18, and 0.03/0.15 [Å] for the neutral, anionic and cationic states of the ds-oligo, respectively.

### 3.2. The Influence of a Ribonucleoside on ds-DNA’s Electronic Properties

The DNA double helix can be regarded as a nanowire in which charge migrates through stacked base pairs [42]. Barton et al. investigated this process and showed that a radical cation/anion can be transferred over a distance of hundreds of angstroms from the point of its induction [43]. DNA damage that occurs on this charge “highway” can disrupt electron or hole migration through ds-DNA [44,45,46,47]. For example, the presence of ^OXO^dG in the ds-oligo constituted the endpoint of radical cations [48]; in particular, the ionisation potential of ^OXO^dG is lower than that observed for canonical nucleotides [49]. On the other hand, ribonucleosides have been found to be more abundant in the genome than other DNA lesions [15,50]. Surprisingly, to the author’s knowledge, their influence on the electronic properties of DNA has not yet been investigated.

Therefore, the following questions arise. (1) Can ribonucleosides influence the ionisation potential and electron affinity of double-stranded oligonucleotides? (2) How does the phosphodiester internucleotide linkage between ribonucleotides and 3′-end-attached 2′-deoxynucleosides affect ds-DNA’s electronic properties?

First, it should be pointed out that the ds-oligo is surrounded by a solvation shell, which stabilises the double helix structure. This layer plays a significant role in the ds-DNA/charge interaction and influences the ionisation potential and electron affinity [51]. From a charge distribution and reorganisation perspective, the nucleus moves much more slowly than electrons (Oppenheimer and Franck–Condon rules) [52]. Therefore, after a one-electron oxidation or reduction process, the electronic properties and charge distribution should be described by the non-equilibrated and equilibrated solvent–solute interaction modes. The above states describe the initial (vertical) ionisation potential and electron affinity of the ds-oligo before the molecule reaches the ground state. According to the Kohn–Sham theory, the energies of HOMO and LUMO (highest occupied molecular orbitals and lowest unoccupied molecular orbitals) correspond to the vertical ionisation potential and electron affinity and can be helpful in its prediction [34]. Table 2 presents the calculated values of the HOMO and LUMO energies.

The analysis of the vertical radical cation and anion in the non-equilibrated state of the discussed ds-DNA fragments revealed the following order of ^NE^VIP: R-DNA > IM-R-DNA > RE-R-DNA > SSB-R-DNA; for ^NE^VEA, the order is SSB-R-DNA > R-DNA > IMc-R-DNA > RE-R-DNA (Table 2). The subsequent equilibrated solvent–solute interaction caused the ^EQ^VIP to decrease by 0.64–0.82 eV; conversely, the ^EQ^VEA increased by 0.65–0.74 eV. As shown in Table 2, the highest ^EQ^VIP and ^EQ^AEA values were obtained for IM-R-DNA (i.e., 6.25 eV and 1.42 eV, respectively). These results indicate that, if the pentaoxyphosphorane is formed and stable enough, it is predisposed to becoming a suitable endpoint for an extra electron. Further relaxation of the structure (the nucleus moves to the molecular ground state) led to adiabatic cation and anion formation. The following order of the calculated AIP and AEA was found: →R-DNA > IM-R-DNA > RE-R-DNA > SSB-DNA and SSB-R-DNA > RE-R-DNA > IM-R-DNA > R-DNA, respectively. These results indicate that the formation of any structure other than the canonical 3′-5′ linkage causes the formation of an endpoint regarding the transfer of a hole (radical cation) or an extra electron (radical anion) through the double helix. Hence, the electronic properties presented in Table 2 correspond well with the theory that replication and repair proteins scan the genome for damage via electron transfer. As expected, the results obtained for canonical ds-DNA show AIP and AEA values of 5.65 eV and 2.09 eV, respectively. This indicates that the preference of ds-DNA for electron and hole capture is lower than that of “disrupted” IM-R-DNA, RE-R-DNA and SSB-R-DNA moieties.

As the only difference between ds-DNA and the investigated ds-oligos is the presence of adenosine (rA3) instead of 2′-deoxyadenosine (A3), the electronic properties of the rA3::T3 base pair were considered. An analysis of the results presented in Table 2 revealed subsequent increases in the vertical ionisation potential (^NE^VIP and ^EQ^VIP) during the reaction presented in Figure 3. The following order was found: SSB-R-DNA > RE-R-DNA > IM-R-DNA > R-DNA. Spatial geometry relaxation led to the achievement of the adiabatic radical cation state, and the AIPs were ranked as follows: R-DNA > SSB-R-DNA > RE-R-DNA > IM-R-DNA. Notably, the presence of a 2′,3′-cycliposhphate group in the rA3::T3 structure, extracted from SSB-R-DNA led to AIP decreases of up to 5.73 eV (Figure 4 and Table 2).

The analysis of the vertical and adiabatic electron affinity of the adenosine–thymidine pair revealed that the ^NE^VEA and ^EQ^VEA were almost at the same level and comparable to those found for the dA::T pair of canonical ds-DNA (Table 2). Surprisingly, when SSB-R-DNA achieved the adiabatic radical anion state, there was a significant increase in the AEA of the rA3::T3 nucleoside pair (i.e., −2.25 eV). As shown in Table 2**,** the presence of the 2′,3′-cyclic-phosphate group led to a 0.49 eV increase in the AEA of rA::T. The above results are in good agreement with experimental data, which indicates that the single-strand break could be the endpoint of hole and electron migration; however, relaxation of its geometry is required.

### 3.3. The Influence of Ribonucleoside’s Presence in ds-DNA on the Charge, Spin and Distribution

**Figure 6:**
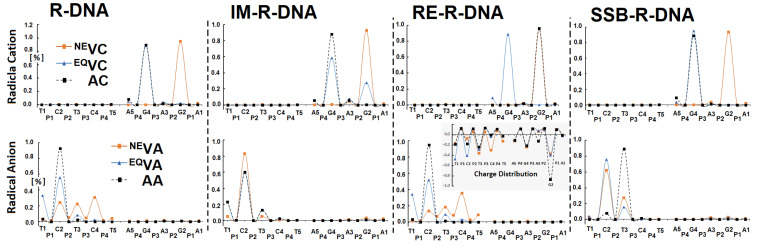
Spin and charge distribution in ds-DNA containing the following at position 3: adenosine R-DNA, adenosine and 2′,5′ phosphodiester linkage RE-R-DNA, adenosine with pentaoxyphosphorane IM-R-DNA, and adenosine with 2′,3′-cyclic-phosphate SSB-R-DNA obtained at the M062x/6-31++G** level of theory in the aqueous phase using the Hirshfeld methodology. The solvent EQ (equilibrated) and NE (non-equilibrated) states were taken into consideration. The raw data are given in Supplementary Materials Table S2.

The electronic properties of the ds-oligos are the outcomes of five nucleosides linked by a phosphate group in each of the single DNA strands. It is important to mention that the external surface of the *ds*-oligo continuously interacts with the solvation (aqueous) layer [51]. Hence, the initial step in the charge migration, HOMO-LUMO and charge/spin distribution, should be considered with reference to the NE/EQ solvent–solute interaction modes [29]. This approach can elucidate the subtle influence of the ribonucleoside’s presence on the charge transfer through the DNA double helix. In these studies, the complete ds-DNA structures have been taken into consideration. As shown in Table S2 and Figure 6, the phosphate backbone does not play a significant role in the spin distribution, which is commonly accepted [54]. The analysis of the Hirshfeld charge distribution showed that, in the non-equilibrated solvent–solute interaction mode, vertical cations accumulate exclusively on one nucleoside; namely, 2′-deoxyguanosine (G2) (Figure 3 and Figure 6). This is opposite to the results obtained from a previous analysis of canonical ds-DNA, where the charge and spin were dispersed over three bases [51]. Solvent–solute equilibration leads to hole migration along the double helix to G4. Only for IM-R-DNA was the spin dispersed over three bases. However, more than 60% of the spin accumulated at the G4 moiety. The radical cation relaxation and adiabatic state achievement by the discussed ds-oligos indicate that 2′-deoxyguanosine (G4) is the endpoint of the hole, except for in RE-R-DNA (Figure 6, Table S2). Surprisingly, phosphodiester bond migration from 3′-5′ to 2′,5′ causes G2 to be more susceptible to radical cation adoption.

**Figure 7:**
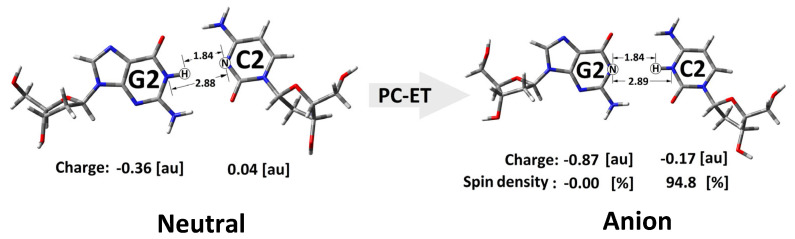
Graphical representation of the proton charge transfer after neutral to adiabatic radical anion relaxation of the dG2::dC2 nucleoside pair isolated from RE-R-DNA.

The situation was similar when an additional electron appeared within the double helix. As shown in Figure 6, in the non-equilibrated state of the vertical radical anion, the spin was not concentrated on a single nucleoside but dispersed over three pyrimidines (C2, T3, and C4) of R-DNA and RE-R-DNA or two (i.e., C2 and T3) of IM-R-DNA and SSB-R-DNA. Relaxation of the solvent–solute interaction leads to the equilibrated state of vertical radical anion formation. The analysis of the spin density reveals the accumulation of unpaired electrons at the C2 moiety at around 60%. The subsequent structure relaxation (ground-state achievement) leads to the adiabatic radical anion state, where the spin is localised exclusively at 2′-deoxycytidine (C2). Surprisingly, single-strand break formation leads to its localisation at thymidine (T3). It should be pointed out that, in each cell, an SSB is formed every 1 or 2 s [55]. The charge analysis of all the discussed cases shows that their location has the same pattern as unpaired electrons (Table S2). Surprisingly, after 3′,5′-phosphodiester bond migration to the 2′,5′-internucleotide linkage (i.e., R-DNA→RE-R-DNA), the negative charge is localised mainly on the 2′-deoxyguanine (G2) moiety, while the spin is found at the 2′-deoxycytidine (C2) of the dG2:::dC2 nucleoside pair (Figure 6, Table S2). The structure analysis of the dG2:::dC2 nucleoside pair (extracted from RE-R-DNA) indicates a proton transfer from the N1 of guanine to the N3 of cytidine (Figure 7).

An analogous situation is not observed for any other nucleoside pair of the discussed ds-oligos. Therefore, it can be postulated that the observed proton-coupled electron transfer can play a significant role in the charge transfer process through a ds-oligo [56].

## 4. Conclusions

Genetic information is continuously exposed to harmful extra- and intracellular factors (ROS, NOS, replication errors and topoisomerase misalignment). On the other hand, the double helix geometry can be disturbed by the incorporation of incorrect sugar nucleosides, which occurs during the replication of genetic material (pol ε/δ incorporates 1 NTP per 1250/5000 nucleotides, and pol α 1 per 625).

Here, for the first time, the influence of a ribonucleotide moiety present in ds-DNA on its electronic properties was theoretically considered at the M062x/6-31++G**//M062x/D95* level of theory in the aqueous phase using the non-equilibrated and equilibrated solvent–solute interaction mode. Moreover, the charge and spin distributions were investigated according to the Hirshfeld methodology. The obtained results are summarised in the following points:

- The stability of the phosphodiester internucleotide bond between the ribonucleotide moiety and DNA decreases after a hole or electron appears in the ds-DNA structure, in comparison to the neutral form.
- Analysis of the electronic properties of an adiabatic radical cation and anion revealed the following order of the calculated AIP and AEA: →R-DNA > IM-R-DNA > RE-R-DNA > SSB-DNA and SSB-R-DNA > RE-R-DNA > IM-R-DNA > R-DNA, respectively. These results indicate that the formation of any structures other than the canonical 3′-5′ linkage causes the formation of an endpoint for the transfer of a hole (radical cation) or an extra electron (radical anion) through the double helix.
- The analysis of the Hirshfeld charge distribution showed that, in the non-equilibrated solvent–solute interaction mode, vertical cations accumulate exclusively on one nucleoside; namely, 2′-deoxyguanosine (G2). Further radical cation relaxation and adiabatic state achievement by the discussed ds-oligos elucidated the 2′-deoxyguanosine (G4) as the endpoint of the hole migration (except in R-DNA, where G2 was found to be more susceptible to radical cation adoption).
- The appearance of an additional electron in the double helix causes the non-equilibrated vertical radical anion state, where the spin is dispersed over three pyrimidines (C2, T3, and C4) of R-DNA and RE-R-DNA or two pyrimidines (C2 and T3) of IM-R-DNA and SSB-R-DNA. Relaxation of the solvent–solute interaction leads to the accumulation of unpaired electrons at the C2 moiety. The subsequent structure relaxation (adiabatic radical anion state) results in the spin being localised exclusively at 2′-deoxycytidine (C2), except for in SSB-R-DNA, where it accumulates at thymidine (T3).

Taking all these results into account, it can be postulated that the introduction of even a single ribonucleoside into ds-DNA can significantly change its electronic properties. Additionally, ribonucleosides likely influence charge transfer through the double helix, subsequently disturbing the process of recognising and repairing DNA lesions. The presence of ribonucleosides in the genome has been observed during disturbed replication and repair processes, understanding their role as important for improving cancer treatments. Moreover, the discussed internucleotide bond stability changes after one-electron oxidation or reduction can support new radiotherapy strategies that are more effective and safer. Therefore, further theoretical and experimental studies are highly warranted.

## Figures and Tables

**Table 1:** The comparative analysis of the rA3::T3 nucleotides pairs: (A) hydrogen bond HB-1, HB-2 lengths in [Å], (B) sugar ring phase [°] and puckering of Adenine 3 and (C) the length of the selected P-O linkage present in the internucleotide bond attached to Adenine 3 moiety in the neutral, anion and cation forms of the discussed ds-oligo, calculated at the M06-2x/D95* level of theory in the aqueous phase.

*ds*-Oligo	Neutral			Cation			Anion		
	HB-1	HB-2 [Å]	P [°]	HB-1	HB-2	P	HB-1	HB-2	P
**DNA**	2.88	2.82	76.4 (^0^T_4_)	2.89	2.85	71.9 (_4_T^0^)	2.93	2.81	74.0 (^0^T_4_)
**R-DNA**	2.90	2.88	156.6 (^2^T_1_)	2.85	2.66	149.0 (^2^T_1_)	2.92	2.93	146.4 (^2^T_1_)
**IM-R-DNA**	2.89	2.84	117.4 (_1_T^0^)	2.90	2.89	191.3 (_3_T^2^)	2.93	2.88	130.0 (_1_T^2^)
**RE-R-DNA**	3.00	2.80	97.7 (^0^T_1_)	3.02	2.84	115.7 (_1_T^0^)	2.90	2.92	127.5 (_1_T^2^)
**SSB-R-DNA**	2.94	2.65	51.8 (_4_T^3^)				2.93	3.03	90.5 (^0^E)
	**Neutral**			**Cation**			**Anion**		
	**P-O2′**	**P-O3′**	**P-O5′**	**P-O2′**	**P-O3′**	**P-O5′**	**P-O2′**	**P-O3′**	**P-O5′**
**DNA**		1.72	1.72		1.72	1.72		1.72	1.72
**R-DNA**	3.45	1.61	1.44	3.54	1.66	1.66	2.91	1.71	1.71
**IM-R-DNA**	1.75	1.77	1.74	1.65	1.74	1.66	1.75	1.77	1.74
**RE-R-DNA**	1.73	2.46	1.72	1.61	3.22	1.60	1.61	3.19	1.60
**SSB-R-DNA**	1.68	1.69	3.33	1.63	1.63	3.57	1.64	1.62	3.30

**Table 2:** The electronic parameters in [eV] of the complete double helix: R-DNA, RE-R-DNA, IM-R-DNA and SSB-R-DNA and rA3::T3 nucleoside pair isolated from the corresponding ds-oligo calculated at the M062x/6-31++G** level of theory in the condensed phase. VIP-vertical ionisation potential, AIP-adiabatic ionisation potential, VEA-vertical electron affinity, AEA-adiabatic electron affinity. Non-equilibrated (NE) and equilibrated (EQ) solvent mode. The details have been shown in the Materials and Methods part. ** indicates isolated nucleosides part (rA3::T3) with 2′,3′-cyclic-phosphate moiety.

*ds*-Oligo	rA::dT Nucleosides Pair						*ds*-oligonucleotides							
	^NE^IP	^EQ^IP	AIP	^NE^EA	^EQ^EA	AEA	^NE^IP	^EQ^IP	AIP	^NE^EA	^EQ^EA	AEA	HOMO	LUMO
**R-DNA**	7.72	6.69	7.04	0.46	1.48	1.44	7.03	6.21	7.41	0.73	1.38	1.76	7.08	0.67
**IM-R-DNA**	7.73	6.70	6.44	0.46	1.48	1.69	6.91	6.25	4.49	0.68	1.42	3.47	6.96	0.67
**RE-R-DNA**	7.74	6.71	6.55	0.43	1.45	1.61	6.84	6.12	3.84	0.62	1.33	4.08	6.90	0.65
**SSB-R-DNA**	7.80	6.78	6.61	0.57	1.58	2.25	6.75	6.09	2.76	0.82	1.62	5.55	6.81	0.78
** SSB-R-DNA	7.84	6.83	5.73	0.57	1.58	2.74								
**DNA** [53]	7.70	6.67	6.63	0.44	1.46	1.48	6.72	6.08	5.65	0.84	1.58	2.09	6.95	0.66

## Data Availability

Data are contained within the article and Supplementary Materials.

## Supplementary Materials

The following supporting information can be downloaded at https://www.mdpi.com/article/10.3390/cells14120881/s1, Table S1a. The energies (in Hartree) of the neural, vertical cation (VCNC) (NE-non-equilibrated), vertical cation (VCEQ) (EQ-equilibrated), vertical anion (VANE), vertical anion (VAEQ), adiabatic cation (AC), and adiabatic anion (AA) of the complete DNA double helix calculated at the M06-2X/6-31++G** level of theory in the aqueous phase. Table S1b. The energies (in Hartree) of the neural, vertical cation (VCNC) (NE-non-equilibrated), vertical cation (VCEQ) (EQ-equilibrated), vertical anion (VANE), vertical anion (VAEQ), adiabatic cation (AC), and adiabatic anion (AA) of the dA3::T3 nucleoside pair skeleton extracted from ds-oligonucleotides calculated at the M06-2X/6-31++G** level of theory in the aqueous phase, respectively. Table S2. Hirshfeld charge (Q) and spin (S) distribution [au] in the shape of a complete double helix: R-DNA, IM-R-DNA, RE-R-DNA, SSB-R-DNA calculated at the M06-2x/6-31++G** level of theory in the aqueous phase. Tabel S3. Values of the torsion angles in [o] of 2-deoxyribose, for the pseudorotation parameter calculation of the dA3::T3 nucleoside pair skeleton extracted from ds-oligonucleotides calculated at the M06-2X/D95* level of theory in the aqueous phase, respectively. The all structures of the discussed ds-oligos are given in *.pdb format.
