# Supplementary material for: The Consequence of the Presence of Ribonucleotide for ds-DNA’s Electronic Properties: Preliminary Theoretical Studies
Source: Cells. 2025 Jun 11;14(12):881. doi: 10.3390/cells14120881 (PMC12190494; doi:10.3390/cells14120881)
Supplement: Supplementary file 1 [file cells-14-00881-s001.zip › Supplementary Materials.pdf]

Supplementarny Materials

The consequence of Ribonucleotide presence on ds-DNA electronic properties: preliminary theoretical studies

Boleslaw T. Karwowski

DNA Damage Laboratory of Food Science Department, Faculty of Pharmacy, Medical University of Lodz, ul. Muszynskiego 1, 90-151 Lodz, Poland; Boleslaw.Karwowski@umed.lodz.pl

Table S1a.

The energies (in Hartree) of Neural, Vertical Cation (VC^{NC}) (NE-non-equilibrated), Vertical Cation (VC^{EQ}) (EQ-equilibrated), Vertical Anion (VA^{NE}), Vertical Anion (VA^{EQ}), Adiabatic Cation (AC), Adiabatic Anion (AA) of complete DNA double helix calculated at the M06-2X/6-31++G** level of theory in the aqueous phase.

ds-oligo	Neutral	NE-VC	EQ-VC	Cation	NE-VA	EQ-VA	Anion
**DNA**	-1762.90444	-1762.62135	-1762.659169	-1762.660848	-1762.920554	-1762.958006	-1762.958957
**R-DNA**	-1838.095658	-1837.811973	-1837.849745	-1837.836781	-1838.112633	-1838.150067	-1838.148558
**IM-R-DNA**	-1838.092663	-1837.808744	-1837.846552	-1837.855937	-1838.109426	-1838.146931	-1838.154669
**RE-R-DNA**	-1838.093854	-1837.809485	-1837.847263	-1837.853024	-1838.109544	-1838.146976	-1838.152904
**SSB-R-DNA**	-1762.904440	-1762.621352	-1762.659169	-1762.660848	-1762.920554	-1762.958006	-1762.958957
****SSB-R-DNA**	-2329.289188	-2329.000913	-2329.038211	-2329.078708	-2329.310275	-2329.347175	-2329.389986

Table S1b.

The energies (in Hartree) of Neural, Vertical Cation (VC^{NC}) (NE-non-equilibrated), Vertical Cation (VC^{EQ}) (EQ-equilibrated), Vertical Anion (VA^{NE}), Vertical Anion (VA^{EQ}), Adiabatic Cation (AC), Adiabatic Anion (AA) of dA3::T3 nucleoside pairs skeleton extracted from *ds*-oligonucleotides calculated at the M06-2X/6-31++G** level of theory in the aqueous phase, respectively

	Neutral	NE-VC	EQ-VC	Cation	NE-VA	EQ-VA	Anion
**DNA**	-12776.342723	-12776.095908	-12776.119271	-12776.134992	-12776.373502	-12776.400715	-12776.419592
**R-DNA**	-12851.595523	-12851.337208	-12851.367359	-12851.323128	-12851.622381	-12851.646377	-12851.660246
**IM-R-DNA**	-12851.531635	-12851.277853	-12851.301829	-12851.366533	-12851.556523	-12851.583893	-12851.659262
**RE-R-DNA**	-12851.523283	-12851.271789	-12851.298557	-12851.382145	-12851.546233	-12851.572164	-12851.673399
**SSB-R-DNA**	-12851.495266	-12851.247184	-12851.271425	-12851.393742	-12851.525532	-12851.554863	-12851.699304

Table S2.

Hirshfeld charge (Q) and spin (S) distribution [au] in the shape of complete double helix: R-DNA, IM-R-DNA, RE-R-DNA, SSB-R-DNA calculated at the M06-2x/6-31++G** level of theory in the aqueous phase.

Neutral form (NF)Vertical Cation (VC^{NC}) (NE-non-equilibrated). Vertical Cation (VC^{EQ}) (EQ-equilibrated). Vertical Anion (VA^{NE}). Vertical Anion (VA^{EQ}). Adiabatic Cation (AC). Adiabatic Anion (AA)

R-DNA													
	NF	VC^{NC}		VC^{EQ}		AC		VA^{NE}		VA^{EQ}		AA	
	Q	S	Q	S	Q	S	Q	S	Q	S	Q	S	Q
**T1**	-0.144	0.000	-0.127	0.000	-0.143	0.000	-0.158	0.026	-0.184	0.329	-0.460	0.034	-0.193
**P1**	0.130	0.000	0.137	0.000	0.130	0.000	0.130	0.014	0.112	0.001	0.121	0.000	0.118
**C2**	0.042	0.001	0.077	0.000	0.046	0.000	0.062	0.240	-0.162	0.554	-0.437	0.924	-0.673
**P2**	0.123	0.000	0.132	0.000	0.123	0.000	0.122	0.049	0.073	0.000	0.115	0.000	0.110
**T3**	-0.174	0.004	-0.167	0.000	-0.167	0.000	-0.174	0.221	-0.367	0.079	-0.257	0.025	-0.227
**P3**	0.134	0.000	0.143	0.000	0.135	0.000	0.137	0.046	0.085	0.000	0.132	0.000	0.134
**C4**	-0.010	0.000	-0.011	0.001	0.020	0.000	0.121	0.303	-0.265	0.019	-0.030	0.000	-0.015
**P4**	0.112	0.000	0.118	0.000	0.113	0.000	0.118	0.016	0.090	0.000	0.111	0.000	0.112
**T5**	-0.031	0.000	-0.024	0.003	-0.017	0.004	-0.016	0.040	-0.087	0.001	-0.034	0.000	-0.030
**A5**	-0.101	0.000	-0.091	0.050	-0.022	0.083	0.011	0.005	-0.116	0.000	-0.102	0.000	-0.103
**P4**	0.114	0.000	0.118	0.000	0.119	0.000	0.121	0.000	0.112	0.000	0.114	0.000	0.112
**G4**	-0.218	0.001	-0.209	0.885	0.546	0.895	0.424	0.009	-0.242	0.001	-0.222	0.000	-0.186
**P3**	0.143	0.000	0.151	0.000	0.149	0.000	0.154	0.000	0.140	0.000	0.143	0.000	0.085
**A3**	-0.112	0.020	-0.069	0.037	-0.055	0.018	-0.066	0.014	-0.138	0.005	-0.127	0.006	-0.117
**P2**	0.284	0.000	0.301	0.000	0.286	0.000	0.288	0.000	0.047	0.000	0.284	0.000	0.277
**G2**	-0.417	0.951	0.321	0.024	-0.395	0.000	-0.418	0.007	-0.198	0.006	-0.446	0.007	-0.509
**P1**	0.105	0.000	0.123	0.000	0.106	0.000	0.107	0.000	0.100	0.000	0.104	0.000	0.108
**A1**	0.019	0.023	0.076	0.000	0.024	0.000	0.035	0.010	0.000	0.006	-0.010	0.004	-0.002

Table S2 cont.

IM-R-DNA													
	NF	VC^{NC}		VC^{EQ}		AC		VA^{NE}		VA^{EQ}		AA	
	Q	S	Q	S	Q	S	Q	Q	S	Q	S	Q	S
**T1**	-0.148	0.000	-0.132	0.000	-0.145	0.000	-0.141	0.051	-0.220	0.228	-0.373	0.228	-0.373
**P1**	0.130	0.000	0.137	0.000	0.130	0.000	0.131	0.003	0.112	0.001	0.122	0.001	0.122
**C2**	0.045	0.001	0.082	0.000	0.057	0.000	0.050	0.829	-0.626	0.600	-0.478	0.600	-0.478
**P2**	0.124	0.000	0.132	0.000	0.124	0.000	0.123	0.001	0.105	0.001	0.116	0.001	0.116
**T3**	-0.185	0.004	-0.175	0.001	-0.174	0.001	-0.174	0.051	-0.236	0.128	-0.311	0.128	-0.311
**P3**	0.133	0.000	0.142	0.000	0.134	0.000	0.135	0.000	0.126	0.000	0.132	0.000	0.132
**C4**	0.001	0.000	0.002	0.000	0.023	0.000	0.094	0.004	-0.006	0.025	-0.026	0.025	-0.026
**P4**	0.112	0.000	0.119	0.000	0.113	0.000	0.115	0.000	0.109	0.000	0.113	0.000	0.113
**T5**	-0.027	0.000	-0.019	0.002	-0.015	0.003	-0.005	0.000	-0.036	0.001	-0.032	0.001	-0.032
**A5**	-0.103	0.000	-0.092	0.053	-0.030	0.055	-0.039	0.000	-0.107	0.000	-0.105	0.000	-0.105
**P4**	0.114	0.000	0.117	0.000	0.117	0.000	0.120	0.000	0.113	0.000	0.115	0.000	0.115
**G4**	-0.229	0.005	-0.219	0.585	0.277	0.881	0.454	0.000	-0.235	0.000	-0.234	0.000	-0.234
**P3**	0.123	0.000	0.131	0.000	0.128	0.000	0.184	0.000	0.121	0.000	0.124	0.000	0.124
**A3**	-0.229	0.045	-0.172	0.077	-0.139	0.059	-0.255	0.012	-0.250	0.003	-0.244	0.003	-0.244
**P2**	0.331	0.001	0.342	0.000	0.336	0.000	0.353	0.000	0.329	0.000	0.331	0.000	0.331
**G2**	-0.330	0.928	0.400	0.277	-0.098	0.001	-0.280	0.028	-0.387	0.007	-0.362	0.007	-0.362
**P1**	0.115	0.000	0.134	0.000	0.118	0.000	0.111	0.000	0.109	0.000	0.115	0.000	0.115
**A1**	0.022	0.016	0.072	0.004	0.041	0.000	0.025	0.021	-0.018	0.005	-0.003	0.005	-0.003

Table S2 cont.

RE-R-DNA													
	NF	VC^{NC}		VC^{EQ}		AC		VA^{NE}		VA^{EQ}		AA	
	Q	S	Q	S	Q	S	Q	Q	S	Q	S	Q	S
T1	-0.145	0.000	-0.128	0.000	-0.144	0.000	-0.118	0.018	-0.175	0.340	-0.471	0.031	-0.181
P1	0.129	0.000	0.136	0.000	0.129	0.000	0.134	0.015	0.112	0.001	0.120	0.000	0.121
C2	0.043	0.001	0.078	0.000	0.044	0.000	0.620	0.133	-0.077	0.517	-0.408	0.948	-0.173
P2	0.123	0.000	0.132	0.000	0.123	0.000	0.133	0.071	0.055	0.001	0.116	0.000	0.120
T3	-0.195	0.005	-0.186	0.000	-0.188	0.004	-0.158	0.184	-0.359	0.090	-0.288	0.014	-0.236
P3	0.133	0.000	0.143	0.000	0.134	0.000	0.136	0.081	0.054	0.000	0.131	0.000	0.132
C4	-0.005	0.000	-0.006	0.000	0.027	0.000	-0.006	0.352	-0.299	0.032	-0.035	0.000	-0.005
P4	0.112	0.000	0.118	0.000	0.113	0.000	0.112	0.028	0.079	0.000	0.111	0.000	0.111
T5	-0.029	0.000	-0.021	0.003	-0.011	0.000	-0.017	0.086	-0.127	0.002	-0.033	0.000	-0.023
A5	-0.106	0.000	-0.097	0.088	0.009	0.000	-0.102	0.006	-0.123	0.000	-0.108	0.000	-0.111
P4	0.114	0.000	0.118	0.000	0.120	0.000	0.115	0.000	0.111	0.000	0.114	0.000	0.115
G4	-0.217	0.001	-0.209	0.887	0.537	0.000	-0.201	0.006	-0.241	0.001	-0.221	0.000	-0.211
P3	0.120	0.000	0.127	0.000	0.127	0.000	0.114	0.000	0.117	0.000	0.120	0.000	0.115
A3	0.084	0.020	0.112	0.021	0.127	0.020	-0.062	0.010	0.064	0.003	0.073	0.002	-0.126
P2	0.097	0.001	0.110	0.000	0.100	0.000	0.145	0.000	0.094	0.000	0.097	0.000	0.127
G2	-0.369	0.956	0.390	0.001	-0.362	0.960	-0.013	0.006	-0.379	0.006	-0.398	0.001	-0.870
P1	0.104	0.000	0.122	0.000	0.104	0.000	0.122	0.000	0.099	0.000	0.103	0.000	0.105
A1	0.008	0.017	0.059	0.000	0.010	0.015	0.048	0.005	-0.005	0.006	-0.022	0.003	-0.013

Table S2 cont.

SSB-R-DNA													
	NF	VC^{NC}		VC^{EQ}		AC		VA^{NE}		VA^{EQ}		AA	
	Q	S	Q	S	Q	S	Q	Q	S	Q	S	Q	S
T1	-0.163	0.000	-0.144	0.000	-0.163	0.000	-0.144	0.029	-0.214	0.035	-0.223	0.001	-0.178
P1	0.130	0.000	0.138	0.000	0.131	0.000	0.130	0.001	0.116	0.001	0.122	0.000	0.124
C2	0.011	0.002	0.050	0.000	0.014	0.000	-0.007	0.621	-0.473	0.758	-0.612	0.080	-0.063
P2	0.115	0.000	0.125	0.000	0.116	0.000	0.116	0.001	0.096	0.000	0.104	0.000	0.114
T3	-0.184	0.003	-0.175	0.000	-0.176	0.000	-0.163	0.272	-0.407	0.156	-0.335	0.896	-0.771
P3	0.133	0.000	0.144	0.000	0.134	0.000	0.137	0.000	0.120	0.000	0.129	0.000	0.132
C4	0.046	0.000	0.041	0.001	0.086	0.000	0.078	0.009	0.033	0.002	0.033	0.013	-0.126
P4	0.125	0.000	0.133	0.000	0.127	0.000	0.115	0.000	0.119	0.000	0.125	0.000	0.111
T5	-0.108	0.000	-0.101	0.004	-0.086	0.004	0.008	0.001	-0.120	0.000	-0.111	0.000	-0.063
A5	-0.081	0.000	-0.071	0.036	-0.009	0.093	-0.001	0.000	-0.089	0.000	-0.083	0.000	-0.106
P4	0.116	0.000	0.119	0.000	0.121	0.000	0.122	0.000	0.114	0.000	0.116	0.000	0.118
G4	-0.230	0.001	-0.223	0.949	0.565	0.890	0.473	0.002	-0.241	0.001	-0.236	0.000	-0.211
P3	0.096	0.000	0.102	0.000	0.102	0.000	0.115	0.000	0.093	0.000	0.096	0.000	0.100
A3	-0.025	0.037	0.014	0.010	0.008	0.014	-0.009	0.024	-0.065	0.014	-0.057	0.005	-0.015
P2	0.207	0.000	0.222	0.000	0.210	0.000	0.138	0.000	0.201	0.000	0.206	0.000	-0.018
G2	-0.324	0.934	0.414	0.000	-0.320	0.000	-0.190	0.025	-0.378	0.021	-0.380	0.005	-0.224
P1	0.105	0.000	0.124	0.000	0.106	0.000	0.084	0.000	0.098	0.000	0.104	0.000	0.094
A1	0.032	0.024	0.089	0.000	0.033	0.000	-0.002	0.014	-0.003	0.011	0.002	0.000	-0.019

Tabel S3

Values of torsion angles in [°] of 2-deoxyribose, for pseudorotation parameters calculation of dA3::T3 nucleoside pairs skeleton extracted from *ds*-oligonucleotides calculated at the M06-2X/D95* level of theory in the aqueous phase, respectively

**ds-oligo**	**Charge**	υ_0	υ_1	υ_2	υ_3	υ_4
**ds-DNA**	Neutral	-39.35	29.77	9.19	-14.37	33.29
	Cation	-37.86	30.48	11.64	-10.83	30.12
	Anion	-40.77	31.91	11.1	-13.26	33.46
**R-DNA**	Neutral	-33.13	48.35	-44.29	26.54	4.07
	Cation	-34.05	47.86	-34.65	24.12	6.31
	Anion	-40.81	51.09	-41.55	19.76	12.76
**IM-R-DNA**	Neutral	-42.21	36.62	-17.28	7.11	30.84
	Cation	-3.72	19.88	-27.5	26.23	-14.27
	Anion	-38.23	39.12	-25.74	4.36	21.39
**RE-R-DNA**	Neutral	-40.69	27.95	-5.45	-18.87	37.24
	Cation	-44.1	38.48	-18.85	-6.17	31.78
	Anion	-39.6	39.72	-24.93	2.7	23.22
**SSB-R-DNA**	Neutral	-19.7	3.86	24.23	-35.81	35.3
	Cation	-21.38	-4.49	25.99	-39.10	38.60
	Anion	-45.91	27.34	-0.4	-26.25	45.8
**Description of dihedral angles**						
υ_0	C4' – O4' – C1' – C2'					
υ_1	O4' – C1' – C2' – C3'					
υ_2	C1' – C2' – C3' – C4'					
υ_3	C2' – C3' – C4' – O4'					
υ_4	C3' – C4' – O4' – C1'					
